# Identifying Patients With a Higher Potential for Recovery Post Left Ventricular Assist Device: A Single-Center Experience

**DOI:** 10.31486/toj.20.0160

**Published:** 2021

**Authors:** Arslan Mirza, Carlos Manuel Romero, Yoshiya Toyoda, Eman A. Hamad

**Affiliations:** ^1^ Temple Heart and Vascular Institute, Temple University Hospital, Philadelphia, PA; ^2^ Department of Internal Medicine, Temple University Hospital, Philadelphia, PA

**Keywords:** *Adult*, *cardiomyopathies*, *circulatory support devices*, *heart failure*, *myocardial remodeling*

## Abstract

**Background:** Few patients with a left ventricular assist device (LVAD) achieve functional myocardial recovery to the point of LVAD explantation. The aim of this study was to highlight some of the hemodynamic and echocardiographic parameters we observed in patients who recovered.

**Methods:** We conducted a retrospective analysis of 7 patients who received the HeartMate II LVAD (Abbott) at Temple Heart and Vascular Institute and subsequently underwent successful explantation following myocardial recovery. We compared baseline characteristics, echocardiographic data, and hemodynamic data.

**Results:** Baseline characteristics of the cohort were as follows: age 51.6 ± 12.0 years, 57.1% male, 42.9% with nonischemic cardiomyopathy, and mean duration of LVAD support of 10.6 months. Comparison of echocardiographic and hemodynamic data (preimplant vs preexplant) revealed the following: left ventricular ejection fraction (%) was 12.8 ± 6.9 vs 52.8 ± 8.1 (*P*=0.0001), right atrial pressure (mmHg) was 12.3 ± 3.4 vs 5.0 ± 4.0 (*P*=0.022), mean pulmonary artery pressure (mmHg) was 36.0 ± 7.8 vs 15.4 ± 7.1 (*P*=0.01), cardiac output (L/min) was 3.6 ± 1.3 vs 5.5 ± 1.8 (*P*=0.004), and cardiac index (L/min/m^2^) was 1.8 ± 0.5 vs 2.7 ± 0.7 (*P*=0.008). Mean LVAD-free survival was 49.1 months. Results were consistent in both ischemic and nonischemic LVAD explants.

**Conclusion:** A potential for LVAD explantation exists in patients with both ischemic and nonischemic cardiomyopathy. Myocardial recovery may be more likely among young patients with nonischemic cardiomyopathy and patients with recently diagnosed ischemic cardiomyopathy. Future prospective studies are needed.

## INTRODUCTION

Heart failure affects nearly 6 million Americans,^1^ is the leading cause of death globally for both men and women, and contributes to approximately 0.3 million deaths per year. An estimated 200,000 patients with advanced heart failure can benefit from advanced heart failure therapies.^2^ Heart transplant is the gold standard for advanced heart failure, but because of the limited number of donor organs, left ventricular assist device (LVAD) therapy plays a major role as a bridge to transplant as well as destination therapy for patients who are not candidates for transplant. Despite great improvement in its design and adverse event profile, LVAD therapy is still limited by many complications such as gastrointestinal bleed, device thrombosis, infection, and device malfunction.^3^ Heart transplant is also associated with complications: a lifelong need for immunosuppression to prevent cardiac allograft rejection, risk of infection, vasculopathy, and organ dysfunction.^4^ Together, these limitations have stimulated research into the feasibility of LVADs as a bridge to structural remodeling and recovery.

Among patients undergoing LVAD implantation, <2% achieve functional myocardial recovery to the point of eventual LVAD explantation.^5^ Numerous investigators—either at single centers or in multi-institutional working groups—have reported successful cases of LVAD removal^6^ and have shared their experiences, including ways to approach patients who may be suitable candidates for LVAD explantation.^7,8^ However, no specific guidelines for LVAD weaning or criteria for explantation exist.^5^ The purpose of our study was to share our experience with recovery and device explantation and list markers that may aid others in predicting the potential for explant.

## METHODS

We conducted a retrospective medical record review of patients admitted to Temple Heart and Vascular Institute between January 2011 and December 2019 who were no longer LVAD dependent. Prior to the start of our study, the Temple Institutional Review Board (IRB) evaluated and approved our protocol and methods. The Temple IRB determined that all the criteria for a waiver of Health Insurance Portability and Accountability Act authorization were met.

As part of our institution's protocol, all patients received a right heart catheterization and transthoracic echocardiogram prior to their LVAD implant. Following LVAD support, patients’ heart failure regimens were optimized using beta blockers, angiotensin-converting enzyme inhibitors or angiotensin receptor blockers, nitrates/hydralazine, and potassium-sparing diuretics when able. Serial echocardiograms and 6-minute walk tests were obtained on all patients at 1 month, followed by 3- to 6-month intervals thereafter as part of routine surveillance.

### Weaning Protocol

Patients showing echocardiographic signs of recovery on routine surveillance were first assessed for explant potential by slightly reducing their LVAD speeds. In the majority of cases, LVAD speeds were reduced in the inpatient setting when patients were not in acute decompensated heart failure. Initial LVAD speeds were not reduced below 8,600 revolutions per minute (rpm). If patients remained asymptomatic at the reduced LVAD speeds, they were then closely followed in the office for worsening symptoms and with surveillance echocardiograms.

Asymptomatic patients with persistent echocardiographic signs of significant recovery—defined as left ventricular ejection fraction (LVEF) >40%—were scheduled for further functional assessment testing using the 6-minute walk test, cardiopulmonary exercise test when able, and an elective weaning study at the cardiac catheterization lab. During a turndown study in the catheterization lab, invasive hemodynamic and echocardiographic parameters were recorded as LVAD speed was slowly turned down by 400 to 500 rpm every 5 to 10 minutes. The speed was reduced to as low as 7,000 rpm before returning it to baseline. The final decision to explant in all cases was done after a multidisciplinary heart failure team evaluation.

Ten patients who were no longer LVAD dependent were initially selected for the study. However, the final analysis included 7 patients who received the HeartMate II LVAD (Abbott) and exhibited significant functional myocardial recovery (LVEF >40%), resulting in successful explant.

Among those excluded, one patient had incomplete recovery (LVEF <40%) but was no longer LVAD dependent because of pump stoppage from driveline fracture. One patient refused explant despite complete recovery. One patient showed signs of initial recovery and had a successful weaning study in the cardiac catheterization lab; however, when taken to the operating room for explant, the patient did not tolerate and failed the final wean.

Data entry and analysis were carried out using SPSS statistical software, version 27 (IBM Corp) and Excel (Microsoft Corp). Paired sample *t* test was used to compare preimplant and preexplant echocardiographic and hemodynamic parameters.

## RESULTS

Table 1 shows basic demographics and outcomes of patients who underwent HeartMate II explant following recovery. Mean age at the time of implant was 51.6 ± 12.0 years. All patients in our study had stage D heart failure as evidenced by their need for inotropic support, short-term mechanical circulatory support, or both prior to LVAD implantation. Three of the patients had ischemic cardiomyopathy. Mean duration of LVAD support was 10.6 months. The mean LVAD-free survival following explant was 49.1 months. Death occurred in 2 patients after a mean LVAD-free survival of 12 months.

**Table 1.** Demographic Characteristics and Outcomes of Patients Who Underwent HeartMate II Left Ventricular Assist Device Explant, n=7

	Patient Number						
Variable	1^a^	2^a^	3	4^a^	5	6^a^	7^a^
Age, years	49	36	67	63	47	60	39
Sex	M	M	F	F	M	M	F
Race	AA	C	C	AA	C	AA	C
Diabetes mellitus	Yes	–	Yes	–	Yes	Yes	–
Coronary artery disease	–	–	–	Yes	Yes	Yes	–
Chronic kidney disease	Yes	–	Yes	–	Yes	Yes	–
Hypertension	Yes	–	Yes	Yes	Yes	Yes	–
Initial heart failure diagnosis, years	>5	<1	1-5	<1	>5	<1	1-5
Etiology of cardiomyopathy	Idiopathic	Idiopathic	Idiopathic	Ischemic	Ischemic	Ischemic	Idiopathic
Implant indication	DT	DT	DT	DT	DT	DT	DT
Preimplant supportive therapy	Tandem	Inotrope, IABP	Inotrope	Inotrope, emergent CABG, IABP	Inotrope, IABP	Tandem	Inotrope
Explant indication	EF recovery, LVAD infection	EF recovery	EF recovery	EF recovery, driveline infection	EF recovery	EF recovery, driveline infection	EF recovery
Preimplant EF, %	10	5	10	20	20	20	5
Preexplant EF, %	60	55	60	60	40	50	45
Duration of LVAD support, months	10	10	12	2	6	8	26
LVAD-free survival, months^a^	77+	69+	16	86+	13	68+	15+
Complications	–	–	Death	–	LVAD reimplant, OHT, death	–	–

^a^As of the writing of this study.

AA, African American; C, Caucasian; CABG, coronary artery bypass graft; DT, destination therapy; EF, ejection fraction; F, female; IABP, intra-aortic balloon pump; LVAD, left ventricular assist device; M, male; OHT, orthotopic heart transplant.

### Improvement in Echocardiographic Parameters

Table 2 shows the difference between preimplant and preexplant echocardiographic parameters in patients with LVAD recovery. We observed a significant improvement in echocardiographic parameters, including LVEF (preimplant 12.8% ± 6.9% vs preexplant 52.8% ± 8.1%, *P*=0.0001), left ventricular end-diastolic internal diameter (preimplant 60.4 mm ± 11.7 mm vs preexplant 42.6 mm ± 5.7 mm, *P*=0.02), and right ventricle diastolic dimensions.

**Table 2.** Comparison of Preimplant and Preexplant Echocardiographic Parameters, n=7

Echocardiographic Parameter	Preimplant, Mean ± SD	Preexplant, Mean ± SD	*P* Value
Heart rate, min	91.7 ± 16.4	69.6 ± 8.9	0.01
Left ventricular ejection fraction, %	12.8 ± 6.9	52.8 ± 8.1	0.0001
Left ventricular end-diastolic internal diameter, mm	60.4 ± 11.7	42.6 ± 5.7	0.02
Left ventricular end-systolic internal diameter, mm	53.4 ± 14.3	33.4 ± 6.2	0.02
Base right ventricular internal diastolic diameter, mm	39.5 ± 6.2	34.8 ± 5.0	0.045
Mid right ventricular internal diastolic diameter, mm	24.3 ± 7.4	18.6 ± 5.6	0.03
Longitudinal right ventricular internal diastolic diameter, mm	77.6 ± 3.7	68.8 ± 5.8	0.006

Note: *P*<0.05 is statistically significant.

### Improvement in Hemodynamic Parameters

Table 3 shows the difference in preimplant and preexplant hemodynamic parameters among patients with LVAD recovery. We observed a significant improvement in right atrial pressure (preimplant 12.3 mmHg ± 3.4 mmHg vs preexplant 5.0 mmHg ± 4.0 mmHg, *P*=0.022), mean pulmonary artery pressure (preimplant 36.0 mmHg ± 7.8 mmHg vs preexplant 15.4 mmHg ± 7.1 mmHg, *P*=0.01), cardiac index (preimplant 1.8 L/min/m^2^ ± 0.5 L/min/m^2^ vs preexplant 2.7 L/min/m^2^ ± 0.7 L/min/m^2^, *P*=0.008), and cardiac output (preimplant 3.6 L/min ± 1.3 L/min vs preexplant 5.5 L/min ± 1.8 L/min, *P*=0.004).

**Table 3.** Comparison of Preimplant and Preexplant Hemodynamic Parameters, n=7

Hemodynamic Parameter	Preimplant, Mean ± SD	Preexplant, Mean ± SD	*P* Value
Right atrial pressure, mmHg	12.3 ± 3.4	5.0 ± 4.0	0.022
Right ventricular systolic pressure, mmHg	56.5 ± 10.2	29.2 ± 11.2	0.067
Right ventricular end-diastolic pressure, mmHg	17.3 ± 2.1	7.6 ± 5.1	0.176
Pulmonary artery systolic pressure, mmHg	54.8 ± 13.5	30.7 ± 9.3	0.036
Pulmonary artery diastolic pressure, mmHg	27.3 ± 5.5	8.9 ± 4.1	0.003
Mean pulmonary artery pressure, mmHg	36.0 ± 7.8	15.4 ± 7.1	0.01
Pulmonary capillary wedge pressure, mmHg	23.3 ± 6.5	7 ± 4.3	0.035
Transpulmonary gradient, mmHg	11.0 ± 6.9	9.8 ± 2.8	0.678
Diastolic pulmonary vascular pressure gradient, mmHg	4.0 ± 3.7	3.2 ± 4.0	0.862
Cardiac output, L/min	3.6 ± 1.3	5.5 ± 1.8	0.004
Cardiac index, L/min/m^2^	1.8 ± 0.5	2.7 ± 0.7	0.008
Pulmonary vascular resistance, Wood units	3.6 ± 1.5	1.9 ± 0.5	0.08
Systemic vascular resistance, dyn·s/cm^5^	1,725.8 ± 779.5	1,248.3 ± 753.9	0.06

Note: *P*<0.05 is statistically significant.

### Improvement in Functional Capacity

Following explant, patients were noted to have improved functional capacity at 6, 12, and 24 months following explant (Figure).

**Figure.**
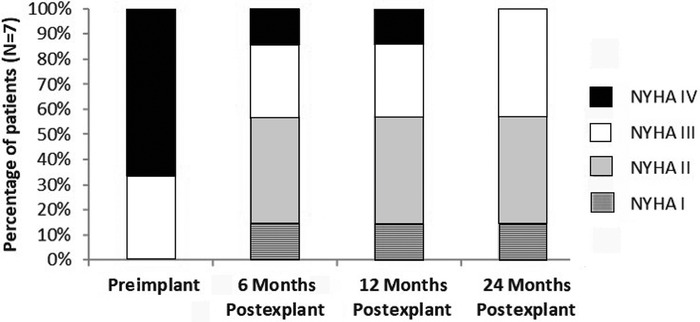
Trend of New York Heart Association (NYHA) functional class from preimplant to 24 months postexplant, n=7.

## DISCUSSION

Our experience with LVAD explant shows that myocardial recovery is possible in patients with both ischemic and nonischemic cardiomyopathy. Although data from LVAD registries show the overall incidence of LVAD explantation to be <2%, centers with dedicated cardiac recovery programs suggest that the rate of cardiac improvement occurs in approximately 15% to 25% of patients with nonischemic cardiomyopathy and in 4% to 5% of patients with ischemic cardiomyopathy.^9,10^ Because of the wide array in the etiologies of myocardial disease, speed of recovery, and proportions of patients who recover, the mechanism for myocardial recovery also likely varies. However, myocardial recovery is believed to be related to various genomic, molecular, cellular, structural, and systemic changes^11^ that occur during chronic left ventricular unloading, resulting in regression of cellular hypertrophy—a phenomenon termed reverse cardiac remodeling.^12^ Clinically, cardiac remodeling manifests as improvement in hemodynamic parameters such as cardiac output, mean pulmonary pressures, left ventricular chamber size, LVEF, and organ perfusion.^13^

Patients in our series were young and more likely to have nonischemic vs ischemic cardiomyopathy (Table 1). Previous studies have shown that young age is associated not only with a higher likelihood of recovery but also with improved survival outcomes.^7,8,14-17^ In most of these studies, the mean age of explanted patients was <40 years.^18^ Interestingly, for the patients in our study, the duration of LVAD support prior to explant was longer in nonischemic vs ischemic patients. Thus, given the lack of donor organs, implanted patients who are <50 years of age and have nonischemic cardiomyopathy should not be rushed into cardiac transplant because they may achieve explant potential after prolonged LVAD support.

We also observed that all of our implanted patients with recent onset heart failure (initial diagnosis <1 year from time of implant) had an LVAD-free survival of >5 years following explant (Table 1). Although limited data are available, previous studies have suggested that patients with shorter durations of heart failure have longer LVAD-free survival time than those with longer durations of heart failure.^7,8,15,16,19^

Explantation, however, does not come without risks. Perioperative mortality of explanted patients is close to 9%, and late mortality is approximately 15%.^18^ However, in patients who are successfully weaned, 5-year freedom from heart failure can reach up to 81%, while survival rates at 1, 5, and 10 years post-LVAD removal have been close to 91%, 76%, and 66%, respectively.^18^

All explanted patients at our center showed normalization of echocardiographic and hemodynamic parameters prior to explant (Tables 2 and 3). While the mean LVEF of our explanted population was >50%, the lowest LVEF in a successfully explanted patient was 40%. Dandel et al showed that a preexplant off-pump LVEF of ≥50% and ≥45% can predict cardiac stability lasting ≥5 years after LVAD removal of 91.7% and 79.1%, respectively.^20^ With each unit of LVEF reduction, the risk of heart failure recurrence is 1.5 times higher.

Although no uniformly accepted weaning criteria exist for device explantation, previous studies have proposed weaning protocols and recommendations. In the Utah Cardiac Recovery study, cardiac recovery was defined as post-LVAD LVEF ≥40% in ≥2 consecutive turndown echocardiograms and no LVEF <40% at later time points (independently from whether the device was eventually explanted).^9^ In contrast, the Berlin study group considered explant to be safe when during repeated off-pump trials performed over several days, the maximum left ventricular end-diastolic diameter was 55 mm, the minimum LVEF was 45%, and the right ventricle size and function remained stable.^8^ Other favorable explant parameters taken into account by prior studies include left ventricular end-diastolic diameter ≤55 mm, left ventricular end-diastolic pressure ≤8 mmHg, cardiac index >2.4 to 2.8 L/min/m^2^, pulmonary artery wedge pressure <13 mmHg, venous oxygen maximum ≥20%, ventilatory efficiency/ventilatory carbon dioxide <34, brachial artery pressure ≥65 mmHg, and a resting heart rate <90/min.^18^

With growing evidence toward cardiac recovery using left ventricular unloading, aggressive management of every patient with end stage heart disease with optimum medical therapy is crucial, especially early after implant in hope for myocardial recovery. Because of a limited number of donor organs and long-term complications associated with both LVADs and transplanted hearts, recovery of the native heart is the most desirable clinical outcome and should be actively sought. The option of transplantation should be used only after recovery of ventricular function has been ruled out. Ruling out recovery prior to transplantation is particularly important in young patients who are unlikely to live a normal lifespan even after a successful heart transplant.^18^

Data from most studies are retrospective with various generations of LVAD devices or include small patient populations, including our study. Future studies should focus on larger prospective populations implanted with the new generation of LVAD devices and should compare various weaning strategies that have been used successfully in the past.

## CONCLUSION

The potential for LVAD explantation exists in patients with both ischemic and nonischemic cardiomyopathy. Future prospective studies are needed to determine the potential of myocardial recovery, especially among young patients with nonischemic cardiomyopathy and patients with recently diagnosed ischemic cardiomyopathy.

## Notes

*This article meets the Accreditation Council for Graduate Medical Education and the American Board of Medical Specialties Maintenance of Certification competencies for Patient Care, Medical Knowledge, and Practice-Based Learning and Improvement.*
